# Professor Austin Flint’s Appeal

**Published:** 1850-12

**Authors:** 


### SELECTIONS AND SYNOPSIS

OF

### IMPORTANT MEDICAL AND SURGICAL INTELLIGENCE CONTAINED IN RECENT MEDICAL JOURNALS.

---

The increasing number of American Medical Journals, and the highly important character of many of their original essays and reports render it difficult to keep pace with the progress of the times, except by abstracts of the main features of the practical papers. This process imposes a great deal of labor upon the editors, but it will be eminently serviceable to the readers of this Journal.

### PROFESSOR AUSTIN FLINT'S APPEAL.

We find the following appeal to the medical profession, in the *Buffalo Medical Journal*, from Prof. Austin Flint, chairman of the standing Committee on *Practical Medicine*, appointed by the American Medical Association, May 1850.

"The undersigned, Chairman of the Standing Committee on *Practical Medicine*, appointed by the American Medical Association, May, 1850, respectfully solicits the coöperation of members of the Medical Profession in furnishing materials for the Annual Report in May, 1851. The duty of this Committee, as defined by the Constitution of the Association, is to "prepare an Annual Report on the more important improvements effected in this country in the management of individual diseases; and on the progress of Epidemics; referring, as occasion requires, to medical topography, and to the character of prevailing diseases in special localities, or in the United States generally, during the term of their service." In order to fulfill the objects thus expressed, the requisite data must be supplied by medical practitioners in different sections of the Union. This is more particularly true with reference to the "*progress of epidemics*" and "*the character of prevailing diseases in special localities.*" Communications, therefore, are particularly desired from persons residing in places in which Epidemics have prevailed, or in which prevailing diseases have been marked by special characters during the present year. Epidemic Cholera and Dysentery are known to have prevailed more or less extensively in different parts of the country during the past summer. Facts bearing upon the features peculiar to the present season, the production, diffusion, mortality, treatment, etc., of these diseases, will be acceptable. It is requested that Communications upon these or any of the subjects coming under the cognizence of the Committee, be transmitted to the undersigned by the first of March, 1851.

"All contributions with which the Committee may be favored, will receive due attention and acknowledgment.

"AUSTIN FLINT.

"BUFFALO, New York, Nov. 1850.

"Editors of Medical Journals will confer a favor by copying the above."

We hope this appeal will be cheerfully and fully responded to by practicing physicians, so that Professor Flint may be able to discharge, properly, the important duties devolving on him. If the profession are alive to the advancement of their true and highest interests, they will do all they can to give usefulness and honor to the labors of the American Medical Association, and we know of no better way, by which they can subserve these ends, than by enabling each standing committee of the Association to do its duty in the most comprehensive manner. By this means the proceedings of the association may be made of the greatest interest, and its deliberations and records will be sure to elevate the profession in public estimation. As the profession rises, the individual members must feel the elevating influence.

The fact cannot be disguised that the proceedings of the Association, at its meeting, in May 1850, have not commanded that respect, nor wielded that healthful influence that attended the earlier efforts of the Association. Instead of a downward tendency, each year should see and feel an upward one; the proceedings of one year, should be an improvement upon those of the preceding year. The Reports of the various committees, generally speaking, seem remarkably meager. The observers appear to have been restricted to a horizon that bounded a narrow field, and beyond that limited sphere, they scarcely looked. This was, to some extent, the fault of the profession; they should have furnished the different committees with useful, important, and interesting facts. The Proceedings of an Association of Physicians, gathered from such an extensive empire as that comprised within the United States; an empire that boasts the possession of almost every climate known to the sun; that contains within itself the means to instruct the whole earth in the science of medicine, should be a national map of medical progress, instructive, full, accurate, and reliable, and with good committees, and a proper interest on the part of the profession, there can be no difficulty in making the proceedings of the American Medical Association all that we have indicated.

There is another point upon which we pray to be indulged. The literature of some of the Reports in the Proceedings of 1850, is not of a very elevated character. We avoided saying anything on this point, in our former remarks, but a writer in the November number of the

*Western Lancet* has been less charry, and has pointed out so many literary blunders, that we think now that attention should be called to this important point. The committees should write good, intelligible English, and constantly bear in mind, that Swift's pithy explantion of a good style—"proper words in proper places"—contains an element for giving honor and respect to the profession, that should not be slighted.

Of Prof. Flint, chairman of the committee on *Practical Medicine*, we wish to say a few words. That he will discharge his duties, to the full extent of his ability, no one who knows him will doubt. There is not in the land a gentleman more fully alive to the interests of the medical profession, nor one more earnestly engaged in advancing those interests. He should be amply and extensively assisted by physicians in every locality in the Union, so that he may be able to present to his medical brethren, a picture of practical medicine, upon which all men may look with a consciousness that it is complete. If the practicing portion of the profession, throughout the Union will send Prof. Flint proper local sketches, we will answer for the work of the artist in chief. B.

